# Neuerungen in der Rechtsschutzorganisation im Immissionsschutzrecht: Auswirkungen der Novelle des Umwelt-Rechtsbehelfsgesetzes 2017 und des Investitionsbeschleunigungsgesetzes 2020

**DOI:** 10.1007/s10357-021-3904-5

**Published:** 2021-11-01

**Authors:** Johannes Saurer

**Affiliations:** Lehrstuhl für Öffentliches Recht, Umweltrecht, Infrastrukturrecht und Rechtsvergleichung, Juristische Fakultät, Universität Tübingen, Tübingen, Deutschland

## Abstract

Das Umwelt-Rechtsbehelfsgesetz 2017 und das Investitionsbeschleunigungsgesetz 2020 haben die gerichtlichen Zuständigkeiten im Immissionsschutzrecht signifikant verändert. Die erstinstanzliche Zuständigkeit der Oberverwaltungsgerichte wurde erheblich erweitert. Für Rechtshelfe Dritter gegen Windenergieanlagen an Land wurde das Entfallen der aufschiebenden Wirkung mit Gesamthöhe von über 50 Metern angeordnet. Der folgende Beitrag analysiert die Neuerungen in der Rechtsschutzorganisation.

#### 1. Einleitung

Der Bundesgesetzgeber hat durch Neuregelungen in der Novelle zum Umwelt-Rechtsbehelfsgesetzes (UmwRG) 2017[1] und im Investitionsbeschleunigungsgesetz 2020[2] die erstinstanzliche Zuständigkeit der Oberverwaltungsgerichte[3] für immissionsschutzrechtliche Streitigkeiten deutlich ausgeweitet. Die Neuregelungen in § 7 Abs. 2 S. 1 UmwRG, § 48 Abs. 1 S. 1 Nr. 3a VwGO und § 48 Abs. 1 S. 1 Nr. 3b VwGO erfassen unter anderem gerichtliche Auseinandersetzungen über Luftreinhaltepläne, Lärmaktionspläne sowie Windenergieanlagen an Land und KWK-Anlagen oberhalb bestimmter Schwellenwerte. In den erfassten Fällen sind verwaltungsgerichtliche Streitigkeiten erstinstanzlich nicht mehr vor den Verwaltungsgerichten, sondern vor den Oberverwaltungsgerichten auszutragen. Der verwaltungsgerichtliche Instanzenzug wird in den erfassten Fällen gegenüber dem regelmäßigen dreistufigen Instanzenzug (vgl. §§ 45 ff. VwGO) um eine (Tatsachen-)Instanz verkürzt. Zugleich legte das Investitionsbeschleunigungsgesetz 2020 mit Einfügung eines neuen § 63 BImSchG das Entfallen der aufschiebenden Wirkung von Rechtsbehelfen Dritter gegen Windenergieanlagen an Land mit Gesamthöhe von mehr als 50 Metern fest. Der folgende Beitrag umreißt zunächst die rechtliche Ausgangssituation der Rechtsschutzorganisation seit den 1970er Jahren (dazu 2). Sodann werden die Neuregelungen zur erstinstanzlichen Zuständigkeit des OVG in § 7 Abs. 2 S. 1 UmwRG, § 48 Abs. 1 S. Nr. 3a VwGO und § 48 Abs. 1 S. Nr. 3b VwGO (dazu 3–5) und die Regelung zum Entfallen der aufschiebenden Wirkung in § 63 BImSchG (dazu 6) analysiert. Zudem werden völkerrechtliche, unionsrechtliche und verfassungsrechtliche Aspekte behandelt (7).

#### 2. Grundzüge der Rechtsschutzorganisation und Entwicklung seit den 1970er Jahren

Die gerichtliche Zuständigkeit für immissionsschutzrechtliche Rechtsstreitigkeiten richtet sich im Ausgangspunkt nach der VwGO. Das BImSchG als Leitgesetz des Immissionsschutzrechts enthält keine spezifischen Regelungen zur gerichtlichen Zuständigkeit. Demgemäß kommt grundsätzlich der dreistufige Instanzenzug der §§ 45 ff. VwGO zum Tragen. Danach ist im Ausgangspunkt das Verwaltungsgericht sachlich zuständig als Eingangsinstanz (§ 45 VwGO), das Oberverwaltungsgericht als Berufungsinstanz (§ 46 VwGO) und das Bundesverwaltungsgericht als Revisionsinstanz (§ 49 VwGO). Bereits Mitte der 1980er Jahre wurde bundesrechtlich eine für das Immissionsschutzrecht wichtige Ausnahme von der regelmäßigen erstinstanzlichen Zuständigkeit der Verwaltungsgerichte geschaffen. Mit dem Beschleunigungsgesetz vom 1985[4] wurde in das Entlastungsgesetz von 1978[5] ein neuer § 9 eingefügt, der die erstinstanzliche Zuständigkeit des Oberverwaltungsgerichts für Rechtsstreitigkeiten über verschiedene Kategorien von Großvorhaben anordnete, darunter gem. § °9 Abs. 1 Nr. 3 die Zuständigkeit für Rechtsstreitigkeiten über „die Errichtung, den Betrieb und die Änderung von Kraftwerken mit Feuerungsanlagen für feste, flüssige und gasförmige Brennstoffe mit einer Feuerungswärmeleistung von mehr als dreihundert Megawatt“.[6] Mit dem 4. VwGO-Änderungsgesetz von 1990[7] wurde diese erstinstanzliche Zuständigkeit des Oberverwaltungsgerichts unverändert in den neu gefassten § 48 Abs. 1 S. 1 Nr. 3 VwGO überführt. In der Sache knüpft die erstinstanzliche Zuständigkeit des Oberverwaltungsgerichts für bestimmte Kraftwerke gem. § 48 Abs. 1 S. 1 Nr. 3 VwGO an die 13. BImSchV (Großfeuerungsanlagen-Verordnung),[8] die bei den Emissionsgrenzwerten nach bestimmten Leistungskenngrößen der erfassten Kraftwerke unterscheidet.[9] Ins-

Prof. Dr. Johannes Saurer, LL.M. (Yale),
Inhaber des Lehrstuhls für Öffentliches Recht, Umweltrecht, Infrastrukturrecht und Rechtsvergleichung,
Juristische Fakultät der Universität Tübingen,
Tübingen, Deutschland

1) Gesetz zur Anpassung des Umwelt-Rechtsbehelfsgesetzes und anderer Vorschriften an europa- und völkerrechtliche Vorgaben v. 29. 5. 2017, BGBl. I S. 1298.
2) Gesetz zur Beschleunigung von Investitionen v. 3. 12. 2020, BGBl. I S. 2694.
3) Gem. § 184 VwGO können die Länder bestimmen, dass die Oberverwaltungsgerichte die Bezeichnung Verwaltungsgerichtshof weiterführen, vgl. § 1 Abs. 1 AGVwGO BW zum Verwaltungsgerichtshof Baden-Württemberg in Mannheim; § 1 Abs. 1 HessAGVwGO zum Hessischen Verwaltungsgerichtshof in Kassel; Art. 1 Abs. 1 BayAGVwGO zum Bayerischen Verwaltungsgerichtshof in München.
4) Gesetz zur Beschleunigung verwaltungsgerichtlicher und finanzgerichtlicher Verfahren v. 4. 7. 1985, BGBl. I S. 1274.
5) Gesetz zur Entlastung der Gerichte in der Verwaltungs- und Finanzgerichtsbarkeit v. 31. 3. 1978, BGBl. I S. 446.
6) Vgl. im zeitlichen Kontext *von Oertzen*, DÖV 1985, 749, 752 f.; zur vorhergehenden rechtspolitischen Diskussion *Ule*, WuV 1983, 23, 23 ff.; *Sendler*, DVBl. 1982, 157, 162 ff.; *Redeker*, DVBl. 1982, 805, 809 f.
7) Viertes Gesetz zur Änderung der Verwaltungsgerichtsordnung v. 17. 12. 1990, BGBl. I S. 2809.
8) 13. BImSchV v. 22. 6. 1983, BGBl. I 719.
9) *Von Oertzen*, DÖV 1985, 749, 753.

besondere differenziert die 13. BImSchV deutlich bei der Festlegung von Emissionsgrenzwerten für Kraftwerke unterhalb und oberhalb von 300 MW Feuerungswärmeleistung.[10] Die Anfang der 1990er Jahre eingeführten Verkürzungen des Instanzenzugs bei Großvorhaben im Kontext der Deutschen Einheit mit erstinstanzlicher Zuständigkeit des Bundesverwaltungsgerichts betrafen nach dem Fachplanungsrecht zulassungsbedürftige Vorhaben, insbesondere Bundesfernstraßen, Bundeswasserstraßen und Eisenbahntrassen.[11] Für den Bereich der immissionsschutzrechtlichen Genehmigungsverfahren führte die Beschleunigungsgesetzgebung der 1990er Jahre etwa Regelungen zur Erleichterung der Heilbarkeit von Verfahrensfehlern auch noch während des gerichtlichen Verfahrens sowie Verfahrenserleichterungen bei der Änderung nach dem BImSchG-genehmigungspflichtiger Anlagen ein.[12]

#### 3. Erstinstanzliche Zuständigkeit des OVG für Rechtsstreitigkeiten um SUP-pflichtige Pläne und Programme

Das erstmals 2006 erlassene UmwRG enthielt in seiner Ursprungsfassung keine Regelungen zur gerichtlichen Zuständigkeit. Mithin galt auch für Verbandsklagen anerkannter Umweltvereinigungen im Anwendungsbereich des UmwRG der dreistufige Instanzenzug gem. §§ 45 ff. VwGO mit dem Verwaltungsgericht als Eingangsinstanz. Mit dem UmwRG-Änderungsgesetz 2017 wurde in § 7 Abs. 2 S. 1 UmwRG erstmals eine spezielle Regelung zur gerichtlichen Zuständigkeit in das Gesetz aufgenommen. Der neue § 7 Abs. 2 S. 1 UmwRG steht im Zusammenhang mit der zeitgleich erfolgten Erweiterung des Anwendungsbereichs des UmwRG auf Entscheidungen über SUP-pflichtige Pläne und Programme gem. § 1 Abs. 1 S. 1 Nr. 4 UmwRG. Nach § 7 Abs. 2 S. 1 UmwRG ist für Rechtsstreitigkeiten zu der neu einbezogenen Kategorie der Pläne und Programme gem. § 1 Abs. 1 S. 1 Nr. 4 UmwRG erstinstanzlich das OVG zuständig. Die Zuständigkeitszuweisung gilt für Hauptsacheverfahren und den einstweiligen Rechtsschutz.[13] Die Reichweite der neu begründeten Zuständigkeit des OVG hängt vom Begriff der „Entscheidungen über Pläne und Programme" i. S. d. § 1 Abs. 1 S. 1 Nr. 4 UmwRG ab. Der Gesetzentwurf zu § 1 Abs. 1 S. 1 Nr. 4 UmwRG begründet die Einfügung der Vorschrift und die damit verbundene Erweiterung des Anwendungsbereichs des UmwRG mit Änderungsbedarf zur vollständigen Umsetzung des Art. 9 Abs. 3 Aarhus-Konvention,[14] der auch im Urteil des BVerwG vom 5. 9. 2013 zur umweltrechtlichen Verbandsklage bei Luftreinhalteplänen[15] deutlich geworden sei.[16] § 1 Abs. 1 S. 1 Nr. 4 UmwRG verweist zur Ausfüllung des Begriffs der „Pläne und Programme" zunächst auf die Begriffsbestimmung in § 2 Abs. 7 UVPG und entsprechende landesrechtliche Vorschriften. Weiter kommt es gemäß § 1 Abs. 1 S. 1 Nr. 4 UmwRG darauf an, ob nach Anl. 5 zum UVPG oder nach Landesrecht eine SUP-Pflicht „bestehen kann". Voraussetzung für die Anwendbarkeit ist, dass im konkreten Einzelfall mindestens eine Vorprüfungspflicht zu bejahen ist.[17] Die Möglichkeit einer SUP-Pflicht genügt.[18] Erfasst werden danach infolge der Nennung in Anl. 5 zum UVPG insbesondere Luftreinhaltepläne gem. § 47 Abs. 1 BImSchG (Anl. 5, Ziff. 2.2.) und Lärmaktionspläne gem. § 47d BImSchG (Anl. 5, Ziff. 2.1.). Maßstab für die SUP-Vorprüfung ist für beide Kategorien gem. § 35 Abs. 1 Nr. 2 UVPG, ob der jeweilige Plan für ein nach Anl. 1 zum UVPG oder nach Landesrecht UVP- bzw. UVP-Vorprüfung pflichtiges Vorhaben einen Rahmen setzt.[19] Die Gesetzesbegründung zur Neuregelung der erstinstanzlichen Zuständigkeit in § 7 Abs. 2 S. 1 UmwRG stellt insbesondere auf die normstrukturelle Vergleichbarkeit von SUP-pflichtigen Plänen und Programmen mit den Rechtsakten im Anwendungsbereich der verwaltungsgerichtlichen Normenkontrollen gem. § 47 VwGO ab,[20] also insbesondere mit Satzungen und Rechtsverordnungen.[21] Die Bezugnahme der Gesetzesbegründung auf den Anwendungsbereich des § 47 VwGO ändert nichts daran, dass sich die Bestimmung des statthaften Rechtsbehelfs im Ausgangspunkt nach allgemeinen verwaltungsprozessualen Regeln richtet. Dies stellt § 7 Abs. 2 S. 2 UmwRG klar, der vorrangig von der Stellung von der Erhebung einer Gestaltungs- oder Leistungsklage bzw. eines Antrags nach § 47 Abs. 1 VwGO ausgeht und nur subsidiär eine entsprechende Anwendung des § 47 VwGO vorsieht.[22] So ist auch nach der Begründung der erstinstanzlichen Zuständigkeit des OVG bei Klagen auf Änderung oder Fortschreibung eines Luftreinhalteplans die allgemeine Leistungsklage statthaft.[23]

Die erstinstanzliche Zuständigkeit der OVG gilt nicht nur für Verbandskläger, sondern auch für Individualkläger, soweit diese nach allgemeinen prozessrechtlichen Maßgaben (insbes. Statthaftigkeit, Klagebefugnis) klageberechtigt sind.[24] Dies ergibt sich aus § 7 Abs. 6 UmwRG, der die Zuständigkeit der OVG auf Rechtsbehelfe von Personen und Vereinigungen nach § 4 Abs. 3 S. 1 Nr. 1 UmwRG erstreckt, d. h. von natürlichen und juristischen Personen gem. § 61 Nr. 1 VwGO und Vereinigungen gem. § 61 Nr. 2 VwGO.[25] In § 8 Abs. 2 UmwRG wurde mit dem UmwRG-Änderungsgesetz eine spezielle Übergangsregelung aufgenommen. Danach gilt die Erweiterung des Anwendungsbereichs gem. § 1 Abs. 1 S. 1 Nr. 4 UmwRG und die korrespondierende erstinstanzliche Zuständigkeit des OVG gem. § 7 Abs. 2 S. 1 UmwRG für alle einschlägigen Entscheidungen, die am 2. 6. 2017 noch keine Bestandskraft erlangt haben. Soweit zeitlich nach der Zuständigkeitsbe-

10) Vgl. § 6 Abs. 1 und Abs. 2 bzw. § 11 Abs. 1 der 13. BImSchV i.°d.°F. v. 2. 5. 2013, BGBl. I S. 1021, 1023, 3754; zuletzt geändert am 19. 6. 2020, BGBl. I S. 132 betreffend Emissionsgrenzwerte für Schwefeloxide für Feuerungsanlagen mit festen bzw. flüssigen Brennstoffen.

11) Vgl. § 5 Abs. 1 Verkehrswegeplanungsbeschleunigungsgesetz v. 16. 12. 1991, BGBl. I S. 2174.

12) Dazu kritisch *Sendler*, Rechtsschutz im Umweltrecht, in: Czajka/Hansmann/Rebentisch (Hrsg.), Immissionsschutzrecht in der Bewährung, 1995, S. 479, 504.

13) *Fellenberg/Schiller*, in: Landmann/Rohmer, Umweltrecht, 86. EL April 2018, § 7 UmwRG, Rdnr. 53.

14) Gesetzesbegründung, BT-Drs. 18/9526, S. 31 f.

15) BVerwG, Urt. v. 5. 9. 2013 – 7 C 21712, NVwZ 2014, 64; zur Erläuterung *Durner*, EurUP 2018, S. 142, 147.

16) Gesetzesbegründung, BT-Drs. 18/9526, S. 23 f. Allerdings ist im Hinblick auf den offenen Wortlaut des Art. 9 Abs. 3 AK, der auf bestimmte Verstöße gegen „umweltbezogene Rechtsvorschriften" abstellt, streitig, ob die enumerative Auflistung in § 1 UmwRG den Anforderungen genügt, vgl. *Guckelberger*, NuR 2020, 217; *Schlacke*, EurUP 2018, 127, 140; *Franzius*, in: Schink/Reidt/Mitschang, UVPG/UmwRG, 2018, § 1 UmwRG, Rdnr. 3.

17) *Fellenberg*, Landmann/Rohmer, § 1 UmwRG, Rdnr. 85.

18) *Guckelberger*, NuR 2020, 217, 219, m.°N.°w. zur Rechtsprechung.

19) Vgl. zur UVP-Pflicht bei einem Luftreinhalteplan BVerwG, Urt. v. 27. 2. 2020 – 7 C 3/19, NVwZ 2020, 1191, 1192.

20) Gesetzesbegründung, BT-Drs. 18/9526, S. 42.

21) Vgl. auch *Durner*, EurUP 2018, 142, 146.

22) *Franzius*, in: Schink/Reidt/Mitschang, UVPG/UmwRG, 2018, § 7 UmwRG, Rdnr. 3; *Bunge*, UmwRG, 2. Aufl. 2019, § 7, Rdnr. 37; *Fellenberg/Schiller*, in: Landmann/Rohmer, Umweltrecht, 86. EL April 2018, § 7 UmwRG, Rdnr. 26 (§ 47 VwGO als „Auffangverfahren").

23) Vgl. VGH Mannheim, Urt. v. 29. 11. 2019 – 10 S 2741/18, juris, Rdnr. 21; OVG Schleswig, Urt. v. 24. 6. 2020 – 5 KN 1/19, juris, Rdnr. 22.

24) *Bunge*, UmwRG, 2. Aufl. 2019, § 7, Rdnr. 45.

25) Näher *Fellenberg/Schiller*, in: Landmann/Rohmer, Umweltrecht, 86. EL April 2018, § 7 UmwRG, Rdnr. 54; dort auch zur Ausgestaltung des Verweises in § 7 Abs. 6 UmwRG hinsichtlich der klagefähigen Rechtsakte.

gründung des OVG Rechtsbehelfe zu Rechtsakten gem. § 1 Abs. 1 S. 1 Nr. 4 UmwRG bei einem Verwaltungsgericht anhängig gemacht werden, hat das Verwaltungsgericht die Sache gem. § 83 VwGO i. V. m. § 17a Abs. 2 S. 1 GVG an das Oberverwaltungsgericht zu verweisen.[26]

#### 4. Erstinstanzliche Zuständigkeit des OVG für Rechtsstreitigkeiten über Windenergieanlagen an Land

Das Investitionsbeschleunigungsgesetz 2020 erweitert die erstinstanzliche Zuständigkeit des OVG durch die Einfügung eines neuen § °48 Abs. 1 S. 1 Nr. 3a VwGO auf Entscheidungen über Rechtsstreitigkeiten betreffend die Errichtung, den Betrieb und die Änderung von Windenergieanlagen an Land mit einer Gesamthöhe von mehr als 50 m. Damit knüpft der Bundesgesetzgeber an die Regelung in Anhang 1 Nr. 1.6 der 4. BImSchV an, die festlegt, dass für Windenergieanlagen in Höhe von mehr als 50 Meter die Pflicht zur Einholung einer Genehmigung nach dem Bundesimmissionsschutzgesetz besteht. Die Zuständigkeitszuweisung an das OVG gilt für das Hauptsacheverfahren und den einstweiligen Rechtsschutz.[27] Das Investitionsbeschleunigungsgesetz enthält keine spezifische Überleitungsvorschrift für die Zuständigkeitsabgrenzung zwischen VG und OVG in zeitlicher Hinsicht. Das OVG Lüneburg hat in einer frühen Entscheidung zum neuen Recht für rechtshängige Klagen nach Inkrafttreten des § °48 Abs. 1 S. 1 Nr. 3a VwGO[28] die Voraussetzungen einer perpetuatio fori nach § 83 S. 1 VwGO i. V. m. § 17 Abs. 1 S. 1 GVG angenommen und hierfür auf die Gesetzgebungsmaterialien abgestellt.[29] Das im Vordergrund stehende Gesetzesmotiv für die Schaffung des § 48 Abs. 1 S. 1 Nr. 3a VwGO ist es, durch Verkürzung des gerichtlichen Instanzenzugs im Rahmen der Energiewende den Ausbau der Windenergie an Land zu beschleunigen.[30] Im bisherigen Verlauf der Energiewende in Deutschland waren Windenergieanlagen an Land die wichtigste Erneuerbare Energien-Technologie. Von 2000 bis Jahr 2020 stieg der Anteil der Stromerzeugung aus erneuerbaren Energien am gesamten Stromverbrauch von etwa 6°% auf 45,4°%.[31] Innerhalb der erneuerbaren Energien dominierte 2020 die Windenergie an Land mit einem Anteil von 41,3°%, gefolgt von Photovoltaik mit 20,2°%, Biogas/Biomethan mit 12,4°% und Offshore-Windenergie mit 10,9°%.[32] Allerdings ist der Zubau an neuen Windenergieanlagen an Land seit 2018 stark zurückgegangen[33] und Probleme bei der weiteren Verwirklichung des Ausbaukorridors für Windenergie an Land (vgl. § 4 Nr. 1 EEG[34]) zeichnen sich ab.[35] Als ein wesentlicher Grund für die Probleme beim weiteren Ausbau der Windenergieanlagen an Land gilt die Vielzahl und Dauer gerichtlicher Verfahren, die gegen die Erteilung von Anlagengenehmigungen geführt werden.[36] Die zügige Durchführung gerichtlicher Kontrollen genehmigter Vorhaben ist ein generell wichtiges Element verlässlicher Rahmenbedingungen für Investitionen in Windenergieanlagen an Land. Zudem ist auch die Verknüpfung des Anlagengenehmigungsrechts des BImSchG mit der Förderung des Erneuerbare Energien-Ausbaus nach dem EEG zu beachten. Gem. § 36 EEG ist im Rahmen von Ausschreibungen für Windenergieanlagen an Land das Vorliegen einer Genehmigung nach dem BImSchG Voraussetzung eines jeden Gebots. Ist ein Zuschlag erteilt worden, so beginnt mit dessen öffentlicher Bekanntgabe gem. § 36e EEG eine Realisierungsfrist für die Windenergieanlage an Land von 30 Monaten,[37] nach deren Ablauf der Zuschlag erlischt. Diese Frist läuft grundsätzlich auch während der Dauer eines gerichtlichen Verfahrens gegen die zugrundeliegende Genehmigung. Dies zeigt § 36e Abs. 2 EEG, der für diesen Fall auf Antrag des Bieters eine Verlängerung dieser Frist um maximal 18 Monate ermöglicht. Mithin drängt auch die Funktionsfähigkeit des Fördersystems des EEG für Windenergieanlagen an Land auf die Vermeidung von überlangen gerichtlichen Verfahren. Zur rechtspraktischen Bedeutung der neuen erstinstanzlichen Zuständigkeit des OVG ist allerdings zu beachten, dass die Dauer gerichtlicher Verfahren nur einer von mehreren Faktoren ist, die gegenwärtig den Ausbau der Windenergie an Land verlangsamen.[38] Weitere wichtige Faktoren sind die Ausgestaltung und Handhabung des behördlichen Genehmigungsverfahrens, Schwächen bei der sozialen Akzeptanz von Windenergieanlagen,[39] die personelle Ausstattung der Gerichte, die Reduktion potenzieller Ausbauflächen durch Festlegung von Mindestabständen zwischen Windenergieanlagen und anderen Formen der Nutzung[40] und Ausschluss von Windenergieanlagen in bestimmten Schutzgebietsgebietstypen[41] sowie die zögerliche Förderung des Weiterbetriebs und des Repowe-

26) Vgl. den Verweisungsbeschluss des VG Sigmaringen, Beschl. v. 21. 8. 2018 – 1 K 1825/18, ZUR 2018, 631, 631; nachfolgend VGH Mannheim, Urt. v. 18. 3. 2019 – 10 S 1977/18, NVwZ 2019, 813; vgl. auch OVG Münster, Beschl. v. 6. 12. 2018 – 8 D 62/18.AK, ZUR 2019, 97; VG Hamburg, Beschl. v. 7. 8. 2018 – 7 K 3876/18, juris, Rdnr. 1; VG Hannover, Beschl. v. 25. 10. 2018 – 4 A 11790/17, juris, Rdnr. 1.

27) *Bostedt*, in: Fehling/Kastner/Störmer (Hrsg.), Verwaltungsrecht, 4. Aufl. 2016, § 80 VwGO, Rdnr. 126.

28) Gem. Art. 11 Abs. 1 des Investitionsbeschleunigungsgesetzes v. 3. 12. 2020 (s. oben Fn. 2) ist dessen Art. 1 (Änderung der VwGO) am Tag nach der Verkündung in Kraft getreten.

29) OVG Lüneburg, Beschl. v. 28. 1. 2021 – 12 MS 6/21, NVwZ 2021, 423, 423 f.; anders VG München, Beschl. v. 18. 2. 2021 – M 28 K 18.4542, BeckRS 2021, 3496 Rdnr. 13 ff.

30) BT-Drs. 19/22139, S. 16.

31) Umweltbundesamt, Erneuerbare Energien in Deutschland: Daten zur Entwicklung im Jahr 2018, Hintergrundpapier, März 2021, Stand 3. 5. 2021, abrufbar unter https://www.umweltbundesamt.de/publikationen/erneuerbare-energien-in-deutschland-2020, S. 7.

32) Umweltbundesamt, Erneuerbare Energien in Deutschland: Daten zur Entwicklung im Jahr 2018, Hintergrundpapier, März 2021, Stand 3. 5. 2021, abrufbar unter https://www.umweltbundesamt.de/publikationen/erneuerbare-energien-in-deutschland-2020, S. 8.

33) Umweltbundesamt, Erneuerbare Energien in Deutschland: Daten zur Entwicklung im Jahr 2018, Hintergrundpapier, März 2021, Stand 3. 5. 2021, abrufbar unter https://www.umweltbundesamt.de/publikationen/erneuerbare-energien-in-deutschland-2020, S. 9.

34) Erneuerbare-Energien-Gesetz v. 21. 7. 2014, BGBl. I S. 1066; zuletzt geändert am 21. 12. 2020, BGBl. I S. 3138.

35) Vor diesem Hintergrund der Vorschlag eines Maßnahmenkatalogs des Bundesministeriums für Wirtschaft und Energie, Stärkung des Ausbaus der Windenergie an Land, 7. 10. 2019, Stand 3. 5. 2021, abrufbar unter https://www.bmwi.de/Redaktion/DE/Downloads/S-T/staerkung-des-ausbaus-der-windenergie-an-land.pdf?__blob=publicationFile.

36) BT-Drs. 19/22139, S. 16.

37) Dazu *Bauer/Kantenwein*, EnWZ 2017, 3, 6.

38) Dazu bereits die Gesetzesbegründung zum Investitionsbeschleunigungsgesetz BT-Drs. 19/22139, S. 16 mit der Einschätzung, dass der Ausbau der Windenergieanlagen an Land „unter anderem" wegen der Vielzahl gerichtlicher Verfahren gegen erteilte Genehmigungen zurückgegangen sei.

39) Vgl. etwa *Barth/Ewen/Schütte/Ziekow*, Konfliktdialog bei der Zulassung von Vorhaben der Energiewende, in: Holstenkamp/Radtke (Hrsg.), Handbuch Energiewende und Partizipation, 2018, S. 583 ff.

40) Dazu *Scheidler*, UPR 2020, 45 ff.; vgl. auch die Neufassung des § 249 Abs. 3 BauGB durch Gesetz v. 14. 8. 2020, BGBl. I S. 1728 mit Öffnungsklausel für landesrechtliche Festlegungen von Mindestabständen zwischen Windenergieanlagen und Wohngebäuden von höchstens 1000 Metern.

41) Vgl. Agora Energiewende, Sofortprogramm Windenergie an Land, 2020, S. 18 f., Stand 3. 5. 2021, abrufbar unter https://www.agora-energiewende.de/veroeffentlichungen/sofortprogramm-windenergie-an-land/.

ring von Altanlagen.[42] Auch sind Schwierigkeiten bei der Umstellung des Fördersystems für Windenergieanlagen an Land auf Ausschreibungen[43] und der strukturelle Konflikt zwischen Windenergieanlagenausbau an Land und Artenschutz[44] zu nennen.

#### 5. Erstinstanzliche Zuständigkeit des OVG für Rechtsstreitigkeiten über KWK-Anlagen

Eine weitere erstinstanzliche Zuständigkeit des OVG begründete das Investitionsbeschleunigungsgesetz 2020 für Rechtsstreitigkeiten über KWK-Anlagen. Nach dem neu eingefügten § 48 Abs. 1 S. 1 Nr. 3b VwGO ist das OVG zuständig für alle Klagen, die Errichtung, Betrieb und Änderung von KWK-Anlagen i. S. d. KWKG ab einer Feuerungswärmeleistung von 50 Megawatt betreffen. Mit dieser Kenngröße knüpft § 48 Abs. 1 S. 1 Nr. 3b VwGO an § 1 Abs. 1 der 13. BImSchV (Großfeuerungsanlagen-Verordnung) an, der für Anlagen mit einer Feuerungswärmeleistung von 50 Megawatt und mehr den Anwendungsbereich der 13. BImSchV eröffnet. In der Entstehungsgeschichte war die Neuregelung im Gesetzentwurf der Bundesregierung vom 4. 9. 2020 noch nicht enthalten gewesen.[45] Jedoch führten die Beratungen im Bundestagsausschuss für Verkehr und digitale Infrastruktur dann zur Aufnahme des neuen § 48 Abs. 1 S. 1 Nr. 3b VwGO in den später verabschiedeten Gesetzentwurf.[46] Hinsichtlich der Zuständigkeit des OVG für Hauptsacheverfahren und einstweiligen Rechtsschutz sowie zu Fragen der zeitlichen Überleitung gilt das zu § 48 Abs. 1 S. 1 Nr. 3a VwGO Ausgeführte entsprechend.[47]

Die Begründung des Bundestagsausschusses für Verkehr und digitale Infrastruktur zur Einfügung des § 48 Abs. 1 S. 1 Nr. 3b VwGO betont, dass mit der Verkürzung des Instanzenzugs „Gerichtsverfahren betreffend Vorhaben beschleunigt werden [sollen], die einen Beitrag zum Klimaschutz leisten können, vor allem, wenn sie mit erneuerbaren Energien betrieben werden oder unvermeidbare Abwärme aus der Verbrennung fossiler Brennstoffe, wie zum Beispiel aus Gaskraftwerken, als Übergangstechnologie nutzen."[48] Wie bei Windenergieanlagen an Land[49] ist auch bei KWK-Anlagen der regelmäßige Abschluss gerichtlicher Verfahren innerhalb eines bestimmten zeitlichen Rahmens eine Funktionsvoraussetzung für das Ausschreibungssystem für Förderungen, das mit dem Anlagengenehmigungsrecht verschränkt ist. Für KWK-Anlagen besteht nach § 18 Abs. 1 KWK-Ausschreibungsverordnung[50] eine Realisierungsfrist von 54 Monaten, deren Verstreichen zum Erlöschen des Zuschlags führt.[51]

#### 6. Entfallen der aufschiebenden Wirkung für Rechtsbehelfe Dritter gegen die Zulassung von Windenergieanlagen an Land

Mit dem Investitionsbeschleunigungsgesetz 2020 wurde auch ein neuer § 63 BImSchG geschaffen, der das Entfallen der aufschiebenden Wirkung für Drittwidersprüche und Drittanfechtungsklagen gegen die Zulassung von Windenergieanlagen an Land mit einer Gesamthöhe von mehr als 50 Metern anordnet. Der Begriff des Dritten in § 63 BImSchG umfasst die qualifiziert betroffene Nachbarschaft einschließlich Grundstückseigentümern, Mietern sowie im Einwirkungsbereich der Anlage berufstätigen Personen, Gemeinden sowie Umweltverbände.[52] Mit dem Kriterium der Mindesthöhe von 50 Metern nimmt der Gesetzgeber wie bei § 48 Abs. 1 S. 1 Nr. 3a VwGO auf die Regelung in Anhang 1 Nr. 1.6 der 4. BImSchV Bezug, die die Genehmigungspflicht nach dem BImSchG begründet. Mit der Einfügung wurde in § 63 BImSchG eine seit mehreren Jahrzehnten unbesetzte Norm des BImSchG wiederbesetzt.[53]

Der neue § 63 BImSchG entfaltet seine Rechtswirkung in der Systematik des Verwaltungsprozessrechts gem. § 80 Abs. 2 S. 1 Nr. 3 VwGO als eine außerhalb der VwGO durch Bundesgesetz getroffene Regelung, die den Suspensiveffekt von Widerspruch und Anfechtungsklage gem. § 80 Abs. 1 S. 1 VwGO entfallen lässt. Die Rechtswirkung des Entfallens der aufschiebenden Wirkung von Drittrechtsbehelfen gegen Windenergieanlagen an Land war bisher rechtspraktisch im Einzelfall durch die Genehmigungsbehörden durch Anordnung der sofortigen Vollziehung gem. § 80 Abs. 2 S. 1 Nr. 4 VwGO herbeigeführt worden. Dies spiegelt sich in einer Vielzahl von Drittklagen gegen Windenergieanlagen an Land, die auch zur alten Rechtslage bereits im Verfahren des einstweiligen Rechtsschutzes geführt worden waren.[54] Der nunmehr durch den Bundesgesetzgeber abstrakt-generell in § 63 BImSchG verankerte Ausschluss der aufschiebenden Wirkung bedeutet eine „gesetzliche Vermutung für das Überwiegen öffentlicher (oder privater) Interessen an der sofortigen Vollziehbarkeit eines Verwaltungsakts gegenüber dem Aufschubinteresse des Rechtsbehelfsführers"[55].

Der Gesetzentwurf zu § 63 BImSchG begründet die Regelung zum Entfallen der aufschiebenden Wirkung mit dem Ziel der Verfahrensbeschleunigung, um im Rahmen

42) Vgl. Agora Energiewende, Sofortprogramm Windenergie an Land, 2020, S. 22 ff., Stand 3. 5. 2021, abrufbar unter https://www.agora-energiewende.de/veroeffentlichungen/sofortprogramm-windenergie-an-land/; s. auch § 21 Abs. 1 Nr. 3a, § 23b Abs. 2 EEG 2021.

43) Vgl. Umweltbundesamt, Erneuerbare Energien in Deutschland: Daten zur Entwicklung im Jahr 2018, Hintergrundpapier, März 2021, S. 9, Stand 3. 5. 2021, abrufbar unter https://www.umweltbundesamt.de/publikationen/erneuerbare-energien-in-deutschland-2020.

44) Vgl. *Köck/Bovet*, ZUR 2018, 579, 581 ff.

45) Gesetzentwurf v. 4. 9. 2020, BT-Drs. 19/22139, S. 5 zur Erweiterung von § 48 VwGO; vgl. aber die vorhergehende Aufführung des § 48 Abs. 1 S. 1 Nr. 3b VwGO im Referentenentwurf zum InvBeschlG v. 2. 8. 2020, S. 2, Stand 3. 5. 2021, abrufbar unter https://www.bmvi.de/SharedDocs/DE/Gesetze-19/investitionsbeschleunigungsgesetz.html?nn=382740, S. 5.

46) BT-Drs. 19/24040.

47) S. o. Abschnitt 4.

48) Vgl. BT-Drs. 19/24040, S. 21.

49) S. o. Abschnitt 4.

50) KWK-Ausschreibungsverordnung v. 10. 8. 2017, BGBl. I S. 3167, geändert am 21. 12. 2020, BGBl. I S. 3138.

51) Das Investitionsbeschleunigungsgesetz 2020 fügte neben § 48 Abs. 1 S. 1 Nr. 3a VwGO und § 48 Abs. 1 S. 1 Nr. 3b VwGO mehrere weitere Tatbestände in § 48 Abs. 1 S. 1 VwGO ein. Zudem wurde eine Reihe weiterer Vorschriften zur Beschleunigung in die VwGO aufgenommen, die im Anwendungsbereich über einzelne Tatbestände des § 48 Abs. 1 S. 1 VwGO hinausgehen, so § 48 Abs. 3 VwGO (Kontinuität eines Spruchkörpers beim OVG), § 101 Abs. 1 S. 2 VwGO (Soll-Vorschrift zur möglichst frühzeitigen mündlichen Verhandlung), § 176 VwGO (befristete Erweiterung des Einsatzes von abgeordneten Richtern auf Lebenszeit und Richtern auf Probe an der Verwaltungsgerichten) sowie § 188a VwGO und § 188b VwGO (Einrichtung von besonderen Kammern und Senaten für Wirtschaftsrecht und Planungsrecht).

52) *Scheidler*, RdE 2021, 189, 91 f.

53) In der Ursprungsfassung des BImSchG v. 15. 3. 1974, BGBl. I S. 721 enthielt § 63 BImSchG mehrere Straftatbestände wegen Verstößen gegen Vorschriften des BImSchG; § 63 BImSchG a.°F. wurde im Zuge des 1. UKG im Gegenzug zur Aufnahme des Umweltstrafrechts in §§ 323 ff. StGB aufgehoben, vgl. Art. 12 Nr. 2 des 18. StrÄndG – Gesetz zur Bekämpfung der Umweltkriminalität – v. 28. 3. 1980, BGBl. I S. 373 (1. UKG).

54) Vgl. beispielhaft OVG Münster, Beschl. v. 1. 4. 2019 – 8 B 1013/18, NVwZ-RR 2019, 857; VGH München, Beschl. v. 23. 1. 2020 – 22 CS 19.2297, NuR 2020, 281; VGH Kassel, Beschl. v. 21. 12. 2015 – 9 B 1607/15, NuR 2016, 476; OVG Greifswald, Beschl. v. 21. 5. 2014 – 3 M 236/13, LKV 2014, 421.

55) *Schoch*, in: Schoch/Schneider, VwGO, Werkstand 39. EL Juli 2020, Bearbeitung 22. EL September 2011, Rdnr. 153.

der Energiewende „die Ausbauziele für Windkraft an Land zu erreichen".[56] Das regelungstechnische Vorbild für § 63 BImSchG ist bis in den Wortlaut hinein § 212a BauGB, der anordnet, dass Widersprüche und Anfechtungsklagen von Dritten gegen die bauaufsichtliche Zulassung eines Vorhabens keine aufschiebende Wirkung haben.[57] § 212a BauGB ist anwendbar bei kleineren Windenergieanlagen mit Gesamthöhe unterhalb der Genehmigungsschwelle des Anhangs 1 Nr. 1.6 der 4. BImSchV von 50 Metern, für die eine Baugenehmigung erforderlich ist.[58]

Eine spezifische Überleitungsregel zur zeitlichen Anwendbarkeit des § 63 BImSchG fehlt. Nach dem mithin anwendbaren allgemeinen Grundsatz des intertemporalen Prozessrechts ist grundsätzlich § 63 BImSchG auch auf Rechtsbehelfe gegen Genehmigungen anwendbar, die bereits vor Inkrafttreten der Vorschrift erteilt worden waren.[59] Aus Gründen des Vertrauensschutzes sollten aber vor Inkrafttreten eingelegte Rechtsbehelfe ihre aufschiebende Wirkung behalten.[60] Hinsichtlich der rechtspraktischen Auswirkungen ist wie bei § 48 Abs. 1 S. 1 Nr. 3a VwGO zu beachten, dass die Dauer gerichtlicher Verfahren nur einer von mehreren Faktoren ist, die den Ausbau der Windenergie an Land potenziell verzögern.[61]

### 7. Völkerrechtliche, unionsrechtliche und verfassungsrechtliche Aspekte

Die Neuregelungen zur Rechtsschutzorganisation im Immissionsschutzrecht durch das UmwRG-Änderungsgesetz 2017 und das Investitionsbeschleunigungsgesetz 2020 sind völker- und unionsrechtlich insbesondere am Maßstab der Aarhus-Konvention (AK)[62] und der dazu ergangenen EU-Rechtsakte[63] zu messen. Denn die mit § 7 Abs. 2 S. 1 UmwRG, § 48 Abs. 1 S. 1 Nr. 3a VwGO und § 48 Abs. 1 S. 1 Nr. 3b VwGO und § 63 BImSchG erfassten Rechtsakte fallen grundsätzlich in den Anwendungsbereich der Aarhus-Konvention. Dabei gilt im Ausgangspunkt, dass die Aarhus-Konvention und die Umsetzungsrechtsakte der EU keine ausdrücklichen Vorgaben zur Ausgestaltung des innerstaatlichen Instanzenzugs machen. Art. 9 AK zum Zugang zu Gericht stellt vielmehr allgemein auf den „Zugang zu einem Überprüfungsverfahren vor einem Gericht oder einer anderen auf gesetzlicher Grundlage geschaffenen unabhängigen und unparteiischen Stelle" (Art. 9 Abs. 1 AK und Abs. 2 AK) bzw. „Zugang zu verwaltungsbehördlichen oder gerichtlichen Verfahren" (Art. 9 Abs. 3 AK) ab. Mithin liegt die Ausgestaltung als zweistufiger Instanzenzug und die Beschränkung auf eine Tatsacheninstanz grundsätzlich in der Hoheit der Mitgliedstaaten. Zu beachten ist zudem Art. 9 Abs. 4 AK, der verlangt, dass die gem. Art. 9 Abs. 1–3 AK von den Mitgliedstaaten zu gewährleistenden Rechtsschutzmöglichkeiten „angemessenen und effektiven Rechtsschutz und, soweit angemessen, auch vorläufigen Rechtsschutz sicher[stellen]" und zudem „fair, gerecht, zügig und nicht übermäßig teuer" sind. Diesen Erfordernissen werden die Neuregelungen in UmwRG-Änderungsgesetz 2017 und im Investitionsbeschleunigungsgesetz 2020 gerecht. Die Verkürzung des Instanzenzugs gem. § 7 Abs. 2 S. 1 UmwRG, § 48 Abs. 1 S. 1 Nr. 3a VwGO und § 48 Abs. 1 S. 1 Nr. 3b VwGO durch erstinstanzliche Zuständigkeit des OVG kann für sich im Hinblick auf das Kriterium des „zügigen Rechtsschutzes" sogar positive Effektive in Anspruch nehmen.[64] Im Hinblick auf die Prozesskosten bestehen auch im Hinblick auf den mit der erstinstanzlichen Zuständigkeit des OVG gem. § 67 Abs. 4 VwGO einhergehenden Anwaltszwang keine Bedenken hinsichtlich des Erfordernisses eines „nicht übermäßig teuren" Verfahrens.[65] Zu § 63 BImSchG gilt, dass Art. 9 Abs. 4 AK zwar die Gewährleistung vorläufigen Rechtsschutzes verlangt, nicht aber einen grundsätzlichen Suspensiveffekt von Rechtsbehelfen. Verfassungsrechtlich ist insbesondere der Maßstab des Grundrechts auf effektiven Rechtsschutz gem. Art. 19 Abs. 4 GG bedeutsam. Nach Art. 19 Abs. 4 GG hat der Gesetzgeber in den Prozessordnungen Vorkehrungen dafür zu treffen, dass „der Einzelne seine Rechte auch tatsächlich wirksam durchsetzen kann und die Folgen staatlicher Eingriffe im Regelfall nicht ohne gerichtliche Prüfung zu tragen hat".[66] Die in § 7 Abs. 2 S. 1 UmwRG, § 48 Abs. 1 S. 1 Nr. 3a VwGO und § 48 Abs. 1 S. 1 Nr. 3b VwGO mit der erstinstanzlichen Zuständigkeit des OVG verbundene Verkürzung des Instanzenzugs und Beschränkung auf eine Tatsacheninstanz hält den Anforderungen des Art. 19 Abs. 4 GG stand. Art. 19 Abs. 4 GG gewährleistet keinen Instanzenzug,[67] sodass eine zweistufige Ausgestaltung wie in den hier erörterten Fällen grundsätzlich verfassungskonform ist. Auch die Regelung des § 63 BImSchG liegt innerhalb des Spektrums der verfassungskonformen Ausgestaltungsmöglichkeiten des effektiven Rechtsschutzes durch den Gesetzgeber. Art. 19 Abs. 4 GG verlangt grundsätzlich nicht, dass Rechtsbehelfe gegenüber Verwaltungsentscheidungen aufschiebende Wirkung entfalten müssen.[68] Ausreichend ist vielmehr die Gewährleistung vorläufigen Rechtsschutzes, um effektiven Rechtsschutz durch Herstellung der aufschiebenden Wirkung herbeizuführen.[69] Dies gilt auch, soweit eine Vielzahl von einfachgesetzlichen Anordnungen des Entfallens der aufschiebenden Wirkung die Grundentscheidung des § 80 Abs. 1 S. 1 VwGO für den Suspensiveffekt von Widerspruch und Anfechtungsklage schwächen.[70]

### 8. Fazit

Die Rechtsschutzorganisation im Immissionsschutzrecht ist im Wandel begriffen. Die Novelle zum Umweltrechtsbehelfsgesetz 2017 und das Investitionsbeschleunigungsgesetz 2020 haben die erstinstanzliche Zuständigkeit der Oberverwaltungsgerichte signifikant ausgeweitet. Nach

56) BT-Drs. 19/22139, S. 25.
57) Zur Vorbildwirkung auch ausdrücklich die Gesetzesbegründung, BT-Drs. 19/22139, S. 25.
58) *Scheidler*, RdE 2021, 189, 190.
59) OVG Münster, Beschl. v. 12.3.2021 – 7 B 8/21, BeckRS 2021, 4819, Rdnr. 4.
60) Dies wurde von einem Teil der Rechtsprechung zu § 212a BauGB vertreten, blieb aber umstritten, vgl. m. w. n. *Kalb/Külpmann*, in: Ernst/Zinkahn/Bielenberg/Krautzberger, BauGB, Stand August 2020, § 212a Rdnr. 18.
61) S. o. Abschnitt 4.
62) Aarhus-Konvention (AK), abgedruckt mit dem Zustimmungsgesetz v. 9.12.2006, BGBl. II S. 1251.
63) Insbes. Richtlinie 2003/35/EG des Europäischen Parlaments und des Rates vom 26.5.2003 über die Beteiligung der Öffentlichkeit bei der Ausarbeitung bestimmter umweltbezogener Pläne und Programme und zur Änderung der Richtlinien 85/337/EWG und 96/61/EG des Rates in Bezug auf die Öffentlichkeitsbeteiligung und den Zugang zu Gerichten, ABl. 2003 L 156, S. 17; Richtlinie 2011/92/EU des Europäischen Parlaments und des Rates vom 13.12.2011 über die Umweltverträglichkeitsprüfung bei bestimmten öffentlichen und privaten Projekten, ABl. 2012 L 26, S. 1.
64) Vgl. *Bunge*, UmwRG, 2. Aufl. 2019, § 7, Rdnr. 33.
65) *Bunge*, UmwRG, 2. Aufl. 2019, § 7, Rdnr. 33.
66) BVerfG, Beschl. v. 5.12.2001 – 2 BvR 527/99, 2 BvR 1337, 1777/00, BVerfGE 104, 220, 231, BVerfGE 96, 27, 39 und öfter.
67) BVerfG. Beschl. v. 5.12.2001 – 2 BvR 527/99, 2 BvR 1337, 1777/00, BVerfGE 104, 220, 231 f.; BVerfG (K), Beschl. v. 9.6.2016 – 1 BvR 2453/12, NVwZ 2016, 1243, 1244 und öfter.
68) BVerfG, Beschl. v. 2.5.1984 – 2 BvR 1413/83, BVerfGE 67, 43, 58; BVerfG (K), Beschl. v. 30.10.2009 – 1 BvR 2395/09, NJW 2010, 1871, 1872 und öfter.
69) BVerfG, Beschl. v. 15.6.1989 – 2 BvL 4/87, BVerfGE 80, 244, 252; BVerfG (K), Beschl. v. 30.10.2009 – 1 BvR 2395/09, NJW 2010, 1871, 1872 und öfter.
70) *Schoch*, in: Schoch/Schneider, VwGO, Werkstand 39. EL Juli 2020, Bearbeitung 22. EL September 2011, Rdnr. 155.

den im Vordergrund stehenden gesetzgeberischen Motiven möchte der Bundesgesetzgeber einerseits mit § 7 Abs. 2 S. 1 UmwRG der normstrukturellen Vergleichbarkeit von SUP-pflichtigen Plänen und Programmen i. S. d. § 1 Abs. 1 S. 1 Nr. 4 UmwRG mit den gem. § 47 VwGO i. V. m. dem Landesrecht der prinzipalen Kontrolle durch die Oberverwaltungsgerichte zugeordneten Handlungsformen der Satzung und der Rechtsverordnung Rechnung tragen. Andererseits zielen die mit dem Investitionsbeschleunigungsgesetz 2020 eingefügten § 48 Abs. 1 S. 1 Nr. 3a VwGO und § 48 Abs. 1 S. 1 Nr. 3b VwGO auf die Beschleunigung der gerichtlichen Kontrolle von Infrastrukturvorhaben mit Bedeutung für die Energiewende (Windenergieanlagen an Land mit einer Gesamthöhe von über 50 Meter, KWK-Anlagen ab einer Feuerungswärmeleistung von 50 Megawatt) durch Beschränkung auf eine Tatsacheninstanz. Infolge der Neuregelungen ist bei der Bestimmung der gerichtlichen Zuständigkeit und des Instanzenzugs im Immissionsschutzrecht deutlich stärker als bisher zu differenzieren. Während es grundsätzlich beim dreistufigen Instanzenzug mit Verwaltungsgericht, Oberverwaltungsgericht und Bundesverwaltungsgericht bleibt, gilt für einen signifikanten Teil immissionsschutzrechtlicher Streitigkeiten nunmehr ein zweistufiger Instanzenzug mit dem Oberverwaltungsgericht als Eingangsinstanz und dem Bundesverwaltungsgericht als Revisionsinstanz. Zudem verankert das Investitionsbeschleunigungsgesetz 2020 in § 63 BImSchG das Entfallen der aufschiebenden Wirkung für Rechtsbehelfe Dritter gegen Windenergieanlagen an Land mit einer Gesamthöhe von über 50 Meter.

**Open Access.** Dieser Artikel wird unter der Creative Commons Namensnennung 4.0 International Lizenz veröffentlicht, welche die Nutzung, Vervielfältigung, Bearbeitung, Verbreitung und Wiedergabe in jeglichem Medium und Format erlaubt, sofern Sie den/die ursprünglichen Autor(en) und die Quelle ordnungsgemäß nennen, einen Link zur Creative Commons Lizenz beifügen und angeben, ob Änderungen vorgenommen wurden.
Die in diesem Artikel enthaltenen Bilder und sonstiges Drittmaterial unterliegen ebenfalls der genannten Creative Commons Lizenz, sofern sich aus der Abbildungslegende nichts anderes ergibt. Sofern das betreffende Material nicht unter der genannten Creative Commons Lizenz steht und die betreffende Handlung nicht nach gesetzlichen Vorschriften erlaubt ist, ist für die oben aufgeführten Weiterverwendungen des Materials die Einwilligung des jeweiligen Rechteinhabers einzuholen.
Weitere Details zur Lizenz entnehmen Sie bitte der Lizenzinformation auf http://creativecommons.org/licenses/by/4.0/deed.de.

Open Access funding enabled and organized by Projekt DEAL.

## Der vernünftige Grund des Tierschutzgesetzes und die Tötung von Tieren in Zoos – ein unerkannter Widerspruch?

**Christian Arleth und Nikklas-Jens Biller-Bomhardt**

*Zoos verstehen sich nicht nur als Stätten der Arterhaltung. Sie sind nach den zwingenden Genehmigungsvoraussetzungen des § 42 Abs. 3 Bundesnaturschutzgesetz (BNatSchG) unter anderem auch verpflichtet, den biologischen Bedürfnissen bei der Haltung der Tiere (Nr. 1), der Pflege der gefangenen[1] Individuen nach dem guten Stand veterinärmedizinischer Praxis (Nr. 2) sowie der Einhaltung der Vorschriften des Tierschutz- und des Artenschutzrechts (Nr. 4) Rechnung zu tragen. Umso erstaunlicher ist es, dass in Zoos Tötungen von dort lebenden Tieren ihre Ursache häufig in monetären oder zuchtgenetischen Beweggründen haben. Mit dem folgenden Beitrag soll die besondere rechtliche Stellung von Zoos im Hinblick auf den im deutschen Tierschutzrecht verankerten Lebensschutz jedes einzelnen Tieres und die sich daraus ergebenden Implikationen für die Prüfung des vernünftigen Grunds der §§ 1 S. 2, 17 Nr. 1 Tierschutzgesetz (TierSchG) anhand verschiedener Fallbeispiele aufgezeigt werden.*

### 1. Einführung

Das mediale Echo war immens, als der Zoo Neumünster zu Beginn der Covid-19-Pandemie im April 2020 verkündete, dass ein Notfallschlachtplan für die in diesem Zoo lebenden Tiere aufgrund der fehlenden Besucher und damit einhergehender fehlender Einnahmen für die Tierhaltung erarbeitet wurde, um festzulegen, welche Tiere als erstes getötet werden müssten. Auch wenn besonders dieser Notfallplan aus Neumünster Aufmerksamkeit erfuhr, ist er kein Einzelfall, sondern wurde von der Zooleitung lediglich vergleichsweise offen kommuniziert.[2] Ebenfalls breite Aufmerksamkeit erfuhr die Anordnung des Zoomanagements in Nürnberg im Jahre 2015 zur Tötung von insgesamt 60 dort lebenden Tieren.[3] Die Tötungen wurden für 33 der Tiere mit Verfütterung der Getöteten an andere Zootiere begründet, für die übrigen 27 damit, dass der Platz im Zoo für diese nicht ausreiche und daher nur diejenigen dersel-

Dr. Christian Arleth,
Juristischer Referent der Stabsstelle des Berliner Landestierschutzbeauftragten, Senatsverwaltung für Justiz, Verbraucherschutz und Antidiskriminierung,
Berlin, Deutschland

Nikklas-Jens Biller-Bomhardt,
Richter in einer Kammer für Bausachen,
Landgericht Frankfurt am Main,
Frankfurt am Main, Deutschland

1) Von „Gefangenschaft" spricht der Gesetzgeber im Bundesnaturschutzgesetz im Hinblick auf die Haltung von Tieren in Zoos selbst, vgl. § 42 Abs. 3 Nr. 7 lit. b) BNatSchG.
2) Vgl. exemplarisch die Berichte bei Redaktionsnetzwerk Deutschland v. 13. 4. 2020, Stand 2. 3. 2021, abrufbar unter https://www.rnd.de/panorama/corona-krise-tierpark-fuhrt-liste-von-tieren-die-getotet-werden-mussten-SGLY2BYZRFB4HDSWGQW-7G3RN74.html, in der Süddeutschen Zeitung v. 15. 4. 2020, Stand 2. 3. 2021, abrufbar unter https://www.sueddeutsche.de/panorama/corona-krise-zoo-notschlachtungen-1.4877112, und dem Spiegel v. 15. 4. 2020, Stand 2. 3. 2021, abrufbar unter https://www.spiegel.de/panorama/coronakrise-zoo-in-neumuenster-erstellt-notschlachtplan-fuer-tiere-a-e045797c-b868-4780-932d-c1c1d2c19105.
3) 14 Hirsche, 10 Ziegen, 7 Schafe, 14 Antilopen, 4 Steinböcke, 7 Rinder, 2 Pferde, 1 Esel und 1 Büffel.
